# Physiological relevance of proton-activated GPCRs

**DOI:** 10.1007/s00424-022-02671-1

**Published:** 2022-03-05

**Authors:** Pedro H. Imenez Silva, Carsten A. Wagner

**Affiliations:** 1 Institute of Physiology, University of Zurich, Winterthurerstrasse 190, CH-8057 Zurich, Switzerland; 2 National Center of Competence in Research NCCR Kidney.CH, Zurich, Switzerland

**Keywords:** pH sensing, Cell signaling, Acid–base balance, Respiration, Kidney, Bone, Inflammation

## Abstract

The detection of H^+^ concentration variations in the extracellular milieu is accomplished by a series of specialized and non-specialized pH-sensing mechanisms. The proton-activated G protein–coupled receptors (GPCRs) GPR4 (Gpr4), TDAG8 (Gpr65), and OGR1 (Gpr68) form a subfamily of proteins capable of triggering intracellular signaling in response to alterations in extracellular pH around physiological values, i.e., in the range between pH 7.5 and 6.5. Expression of these receptors is widespread for GPR4 and OGR1 with particularly high levels in endothelial cells and vascular smooth muscle cells, respectively, while expression of TDAG8 appears to be more restricted to the immune compartment. These receptors have been linked to several well-studied pH-dependent physiological activities including central control of respiration, renal adaption to changes in acid–base status, secretion of insulin and peripheral responsiveness to insulin, mechanosensation, and cellular chemotaxis. Their role in pathological processes such as the genesis and progression of several inflammatory diseases (asthma, inflammatory bowel disease), and tumor cell metabolism and invasiveness, is increasingly receiving more attention and makes these receptors novel and interesting targets for therapy. In this review, we cover the role of these receptors in physiological processes and will briefly discuss some implications for disease processes.

## Introduction

Acid–base balance is mainly maintained by the action of the kidneys and lungs that respond to changes in acid–base conditions by controlling the levels of buffers in the blood and by pacing the elimination of acids and bases. Moreover, the control of breathing pattern is performed by the central nervous system, which in turn is highly acid–base sensitive. Organs like the kidneys and brain require mechanisms that constantly detect systemic pH levels to support the control of acid–base balance. Therefore, dedicated pH-sensing mechanisms are present in these organs and play a crucial role protecting the organism from acid–base disorders. The importance of maintaining acid–base balance is illustrated by the multitude of functional alterations observed under conditions of acidemia or alkalemia. During acute acidemia, increased vasodilatation in brain and other organs along with hypotension is observed, cardiac output and contractility is reduced, resistance to catecholamines and insulin occurs, leucocyte and lymphocyte function is suppressed while the secretion of various interleukins is stimulated. Cellular energy production is decreased despite reduced affinity of oxygen to hemoglobin. Cellular apoptosis is stimulated. Severe acidemia may lead to deterioration of the mental status suggesting impaired neuronal functions. In more chronic states of acidemia, bone disease may occur, skeletal muscle is wasted, hepatic albumin production is reduced, glucose metabolism is impaired, and electrolyte balance disturbed. In children, growth retardation may occur, while in patients with kidney disease, loss of renal function may be accelerated [57, 102]. Likewise, alkalemia has many often opposing effects including arterial vasoconstriction, reduced blood flow, and hypertension; increased neuromuscular excitability (only in part due to reductions in ionized calcium); arrhythmias; changes in electrolytes with secondary effects on the brain, muscle, kidneys, and cardiovascular system, and neurological signs including dizziness, nausea, stupor, and coma [19, 54, 63].

As discussed below, some of these functional disturbances can be directly linked to pH-sensing proteins including also the G protein–coupled receptors OGR1, GPR4, and TDAG8. Although multiple molecules of biological relevance can have their function modified by alterations in H^+^ concentration, some groups of proteins can detect alterations in extracellular pH in the physiological range and trigger intracellular responses. Therefore, pH influences cell activity not only by directly changing structure of proteins, protein–protein interactions, biochemical reactions, and bioavailability of molecules and metabolites, but also by interacting with membrane proteins controlling in a regulated manner intracellular activities. The identification of proton sensors and proton-modulated mechanisms changed the view of how cells locally detect acid–base status. These receptors belong to different types of proteins, in which the most well-studied are as follows: (1) ion channels, such as acid-sensing ion channels (ASIC) [8], the renal outer medullary K^+^ channel (ROMK) [101], TWIK-related acid-sensitive K^+^ channel (TASK) [6], and transient receptor potential channels (TRP) [87]; (2) tyrosine kinases, such as insulin receptor–related receptor (IRRR) [14]; and (3) G protein–coupled receptors (GPCRs), the topic of this review. There are also additional types of acid–base sensing molecules, such as the bicarbonate/CO_2_-sensing protein receptor protein tyrosine phosphatase (RPTPgamma) [4], the soluble adenylyl cyclase (sAC) [106], or the pH-sensitive Pyk2 kinase [69].

Of note, the range of pH to be considered physiological can vary substantially between different organs and compartment. While the pH of most extracellular fluids is in the range between pH 7.3 and 7.5, it can be substantially more acidic or alkaline in specific compartments such as the synaptic cleft, the epididymal lumen, along the renal tubule, in bone, or along the axis of the gastrointestinal tract to name a few examples. This alone may suggest that multiple mechanisms must exist that can sense pH in specific ranges.

A protein subfamily belonging to the class A (or rhodopsin-like family) of G protein–coupled receptors (GPCRs) detects changes in extracellular pH in the range of pH 6.5 to 7.5. This subfamily of proteins is composed of four members: G protein-coupled receptor 4 (GPR4 or Gpr4), T-cell death–associated gene 8 (TDAG8 or Gpr65), ovarian cancer G protein–coupled receptor 1 (OGR1 or Gpr68), and G protein–coupled receptor 132 (G2A or Gpr132). However, the role of G2A in pH sensing is unclear as it mostly lacks sensitivity to protons [100]. G2A when cotransfected with OGR1 in HEK293T cells forms a heteromer with high sensitivity to extracellular pH alterations [39]. However, a biological relevance of this heteromer has not been demonstrated. Moreover, it appears that GPR132 diverged early in the evolution of this subfamily, followed by *Gpr65*, and then *Gpr4* and *Gpr68* [107]. Since G2A probably lacks sensitivity to protons and its role in pH-dependent physiological responses is not clear yet, this review is focusing only on the role of GPR4, TDAG8, and OGR1. It should be also noted that multiple other GPCRs may be modulated positively or negatively by protons and GPCRs other than those covered in this review may also function as protons sensors. For example, Kopolka et al. demonstrated that the adenosine A2a receptor can be activated solely by H^+^ [52]. Also, the affinity of the calcium-sensing receptor (CaSR) to calcium is modulated by extracellular protons [17]. Therefore, some GPCRs are activated by H^+^ alone (no matter whether H^+^ is the only (known) ligand or one of several possible ligands), while in other GPCR families, H^+^ is a (strong) modulator of receptor function [43]. For this reason, we use in this review the term “proton-activated GPCR” instead of “proton sensing GPCR” to distinguish between these different modes of action. The current understanding of these receptors is that they require protonation to function while they are modulated or co-activated by other stimuli, at least in the case of OGR1. Therefore, they function as dedicated membrane receptors for protons.

In this review, we are summarizing current knowledge and concepts about the role of the proton-activated GPCRs OGR1, GPR4, and TDAG8 focusing on normal physiology as this aspect has not received much attention. The pharmacology of these receptors and their implication into pathophysiological states has been recently reviewed elsewhere [47, 114].

## Proton-activated receptors

**Fig. 1:**
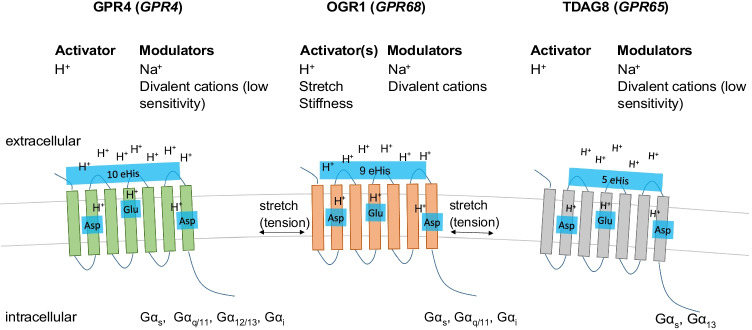
Summary of activators and modulators of proton-activated GPCRs and the Gα subunits coupled to these receptors. Extracellular histidine residues (eHis) and a buried triad of amino acids have been proposed as the mechanisms underlying proton sensitivity in these receptors. Asp, aspartic acid; Glu, glutamic acid

The genes encoding human GPR4, OGR1, and TDAG8 were first identified in the 1990s [62, 78, 148], although murine *Gpr65* had already been identified when the human sequence was cloned [13]. However, their function(s) and ligand(s) had remained elusive. At the beginning of the 2000s, lipids, such as sphingosylphosphorylcholine, lysophosphatidylcholine, and psychosine, were proposed as OGR1 and GPR4 ligands. However, these papers were all retracted because of concerns about the validity of data [51, 150, 161]. A few years later, the activation of OGR1, GPR4, and TDAG8 by extracellular H^+^ was demonstrated [75, 134]. Proton sensitivity is achieved by proteins via changes in the charge of amino acids by protonation or deprotonation. Only a few amino acids are protonated at pH values ranging from 5 to 7.4, such as histidine, aspartic acid, arginine, lysine, and glutamic acid [107]. Research on proton sensing by GPCRs has mostly focused on the role of histidine residues [38, 75, 100, 107]. Several histidine residues are present in the extracellular loops of TDAG8, GPR4, and OGR1, but only few in G2A, which could explain the low pH sensitivity of this receptor [100]. Mutation of single or multiple histidine residues in OGR1 reduces or abolishes pH sensitivity demonstrating that these residues are critical for receptor activation [75, 85]. However, more recently, Rowe et al. proposed that proton sensitivity is primarily derived from a triad of buried residues composed of an aspartic acid and two glutamic acid and not by extracellular histidine residues, which would potentially have other roles, such as in the interaction with other peptides or ions [107]. Indeed, multiple ions and conditions can modulate or stimulate proton-activated GPCRs. Sodium and divalent cations can allosteric modify the activation of GPR4, OGR1, and TDAG8 by H^+^ (although GPR4 and TDAG8 have low sensitivity to divalent cations) [38, 107] (Fig. 1). Whether the modulation of proton-activated GPCRs by these ions in vitro has in vivo relevance is unknown.

## OGR1

A seminal paper by Ludwig et al. showed that extracellular H^+^ stimulates OGR1 which couples to the Gα_q/11_ α subunit stimulating phospholipase C and elevating intracellular Ca^2+^ concentration [75]. However, as seen for the other two proton-activated GPCRs, OGR1 can signal via multiple Gα subunits and downstream signaling pathways. For example, OGR1 also couples to Gαs and Gα_i_ [97, 113]. Additional examples are shown in Table 1.

**Table 1:** Summary of signaling pathways linked to the activity of OGR1, GPR4, or TDAG8 in various cells and cell systems

Receptor	Cell type	Stimuli	Gα subunit	Downstream signalling	Reference
OGR1 (*Gpr68*)	Hamster fibroblasts (CCL39)	pH ~ 5.5–8.5 (min)	Gα_q/11_	IP/Ca^2+^_i_	[75]
	Mouse neuroblastoma cells (N1E-115 cells)	pH ~ 6.1–7.6 (min–2 h)	Gα_q/11_	cGMP/IP/Ca^2+^	[55]
	HEK293 and human lung fibroblasts	pH 6.4, 7.4, and 8.0; sulazepam and lorazepam (< 1 h)	Gα_s_	cAMP/PKA/p-VASP	[97]
	HEK293 and human lung fibroblasts	pH 6.8, 7.4, and 8.0; lorazepam (< 1 h)	Gα_q_ and Gα_i_ (likely)	Ca^2+^/p-ERK	[97]
	Breast cancer cells (MCF7)	OGR1 overexpression	Gα_12/13_	Rho, Rac1	[68]
	Prostate cancer cells PC3	OGR1 transfected vs non-transfected	Gα_i_		[113]
	Pancreatic cancer–associated fibroblasts	pH 6.4–7.4 (< 1 h)		cAMP	[143]
	Human airway smooth muscle cells	pH 6.4–8 (< 1 h)		Ca^2+^, cAMP, p-ERK, p-VASP	[111]
	Mouse hippocampal slices	pH 6.0 vs 7.4 (1 h)		p-PKA, p-AKT, p-Y	[159]
	Endothelial progenitor cells	pH 6.4 vs 7.4) (> 1 h)		p-STAT3, VEGFA	[94]
	Intestine (Caco2)	pH 6.6–7.8 (> 24 h)		IRE1α/JNK	[77]
	HEK293T	pH 6.5; shear stress (2 Pa) (2 min)		Ca^2+^; PLC/Ca^2+^	[147]
	Rat endplate chondrocytes	pH 6.4 vs 7.4 (min–hrs)		Ca^2+^/calpain, calcineurin	[155]
	Goat mammary epithelial cells	OGR1 silencing		p-AKT, p-mTOR	[160]
TGDA8 (*Gpr65*)	Chinese master ovary cells (CHO) and hepatoma (RH7777)	pH ~ 6–8 (< 1 h)	Gα_s_	cAMP	[134]
	HeLa cells	pH 6.5 vs 7.5 (< 1 h)		cAMP	[64]
	Human keratinocytes	CO_2_ 15% vs 5% + UVB (24 h)		cAMP, I-κBα, p65	[112]
	Mouse microglia	pH 6.8 vs 7.6 + LPS (< 1 h)		cAMP, p-ERK, p-JNK	[48]
	CD14^+^ monocytes from IBD patients carrying *Gpr65* mutation	pH 6.5 vs 7.6		cAMP	
	Monocyte (U937)	pH ~ 6.4 vs 7.4 (hrs)	Gα_13_	Rho/c-myc	[50]
	Post-ischemic rat primary cortical neurons	TDAG8 pharmacological agonism (6 h + ischemia)		p-AKT, p-CREB	[76]
	Mouse lymphoma cell (WEHI7.2)	pH 6.5 vs 7.5 (< 1 h)		p-ERK, p-CREB	[108]
GPR4 (*Gpr4*)	HEK293	pH ~ 5.5–8.5	Gα_s_	cAMP	[75]
	Hepatoma (RH7777)	pH ~ 6.5–8 (< 1 h)	Gα_s_	AC/cAMP	[122]
	HEK293 cells	pH ~ 6.2–7.8 (< 1 h)	Gα_s_	cAMP	[73]
	Human umbilical vein endothelial cells	pH 6.4, 7.4, and 8.4 (< 1 h)	Gα_s_	cAMP/EPAC	[10]
	HEK293	pH ~ 6.2–7.8 (< 1 h)	Gα_q/11_	IP/NFAT	[122]
	HEK293	pH 7.15 vs 7.6 (< 1 h)	Gα_13_	Rho/SRE	[122]
	Human umbilical vein endothelial cells	pH 6.4 vs 7.4 (hrs)	Gα_12/13_	Rho/ROCK/MLCK	[58]
	Colorectal carcinoma (HCT116, HT29, SW620, SW480)	GPR4 silencing		RhoA/LATS/YAP1 (hippo)	[154]
	Human trophoblast cell line (HTR8/SVneo)	GPR4 silencing and overexpression		p-MEK, p-ERK	[99]

OGR1 can also be allosterically activated by multiple benzodiazepines, such as lorazepam, diazepam, desmethyldiazepam, and clobazam, but with different efficacies [37, 97]. Using the putative lorazepam site in OGR1 as a starting point, Huang et al. performed a sequence of computational screens and in vitro tests and designed the compound ZINC67740571 and named it ogerin [37]. This compound shows a stronger allosteric effect than lorazepam and causes an elevation of pH-dependent cAMP release while it reduces pH-dependent calcium release in HEK293T cells transfected with *Gpr68*. In vivo, ogerin was shown to affect fear conditioning in mice.

Regulation of GPCR activity often involves negative feedback mechanisms that reduce the receptor responsiveness upon activation. This process, also known as desensitization, occurs through uncoupling between GPCR and G protein or decreased availability of the receptor, which is regulated by internalization, degradation, or reduction of its expression levels. Information regarding proton-activated GPCR desensitization is still scarce. Ludwig et al. did not find evidence for the desensitization of OGR1 in the CCL39 hamster fibroblasts cell line [75]. However, activation of OGR1 by low pH and lorazepam led to desensitization of OGR1 signaling in HEK293T, HEK293, and human airway smooth muscle cells (HASM) [39, 91]. Lorazepam and/or pH 6.8 caused internalization of a HA-tagged OGR1 in HEK293 within 5 min. In HASM cells, Nayak et al. assessed desensitization via analysis of the intracellular signaling downstream of endogenous OGR1 [91]. Authors observed that intracellular calcium elevation in response to lorazepam and low pH was reduced upon restimulation, which did not happen when a non-OGR1 stimulating drug, methacholine, was used. These results suggest that OGR1 coupled to Gα_q_ may be functionally desensitized in HASM cells. Phosphorylation of p42/44, an intracellular signaling step downstream of Gα_q_, was inhibited by lorazepam and low pH within 30 min, but by lorazepam alone only after 24 h incubation. On the other hand, restimulation with sulazepam and low pH reduced phosphorylation of VASP, a molecule downstream of OGR1/Gα_s_. Thus, the activity of OGR1 may be controlled by desensitization by protons and allosteric agonists. This process has different kinetics depending on the agonist and the coupled Gα protein.

In contrast to these findings, Tan and colleagues reported that medium with pH 6.6 does not cause internalization of OGR1 in leucocytes, while pH 7.7 causes partial internalization [119]. Reacidification of the medium caused partial reinsertion of OGR1 in the plasma membrane. Authors attributed the differences to the work by Huang et al. (Nayak et al. was not published at that time) to the method used to detect OGR1 expression.

In contrast with the direct regulation of GPCRs via desensitization, downstream signaling can be inhibited via regulators of G protein signaling (RGS). These proteins most typically terminate GPCR-mediated signal transduction by accelerating the Gα intrinsic hydrolytic rate from GTP to GDP and by reverting the heterotrimeric G protein to its inactive state. Most likely proton-activated GPCR signaling is regulated by multiple RGS, but only little is known about the detailed role of these proteins in the regulation of proton sensors. Airway surface liquid pH plays an important role in the physiology and pathophysiology of the lungs [156]. Both low pH and inflammation can induce hypersecretion of mucins, glycoproteins that form the airway mucus [30, 72]. The human bronchial epithelial cell line (16HBE) secretes a mucin named MUC5AC when exposed to medium with low pH [72]. This process is OGR1-dependent and is inhibited by RGS2. Acidity levels also play a pivotal role in the activity of osteoblasts and osteoclasts. The potential of RGS in the control of pH sensing is further illustrated by in vitro experiments using human umbilical vein endothelial cells (HUVECs) transfected with Rho guanine nucleotide exchange factor 1 (p115-RhoGEF, *Arhgef1*). This protein functions as an RGS by inhibiting Gα_12/13_ signaling, and it inhibits GPR4-mediated paracellular gap formation in HUVECs [58].

Although protons are recognized as the main activators of OGR1, GPR4, and TDAG8, membrane stretch may be essential for OGR1 activity [138]. Experiments performed with cell lines from multiple organs demonstrated that the pH-dependent activation of OGR1-Gαq is also dependent on the stiffness of the cell culture substrate and cell shape [138]. Moreover, using stretchable membranes, authors identified that cell stretch also activates OGR1 signaling, which is blunted if actin polymerization is inhibited. These results are in agreement with in vivo experiments performed by another group, in which shear stress was shown to activate OGR1 in mouse endothelial cells [147]. In this study, OGR1 was mainly found in small diameter arterioles of different organs like the intestine, brain, pancreas, and liver. Deletion of OGR1 not only eliminated the increase in intracellular calcium in response to flow in primary microvascular endothelial cells, but it also attenuated dilation and remodeling of third order mesenteric arteries in response to elevated blood flow [147]. Interestingly, the increase in intracellular calcium induced by shear stress observed in a breast cancer cell line was mostly absent at pH values below 6 and above 8**,** demonstrating that mechanosensing via OGR1 requires pH values in the same range as for its activation by protons [147]. Therefore, both works show that OGR1 is a coincidence sensor for pH and mechanostimuli, e.g., both stimuli are concomitantly essential for the normal activity of OGR1, a seemingly unique feature among GPCRs.

## TDAG8

While extensive research on the role of GPR4 and TDAG8 in physiology and pathophysiology has been done, they are understudied when it comes to investigate their basic properties, such as their regulation at molecular level and downstream signaling.

TDAG8 stimulates cAMP formation and protein kinase A activation via Gα_s_ [134], but it can also couple to Gα_13_ [50]. Initial in vitro data have been supported by experiments in vivo. Mouse type I astroglial cells from wild-type mice show a progressive increase in cAMP production in response to acidification, which is mostly blunted in astroglia from *Gpr65*-deficient mice [48]. Further downstream from these events, activation of TDAG8 has been shown to influence multiple intracellular pathways (Table 1). The same astroglial cells from *Gpr65*-deficient mice lack the reduction in phosphorylation levels of extracellular signal-regulated kinase (ERK1/2 or MAPK) and c-Jun N-terminal kinase (JNK) in response to concomitant exposure to acidification and lipopolysaccharide (LPS) [48]. Likewise, pharmacological stimulation of TDAG8 in rats post-cerebral ischemia induced phosphorylation of AKT and CREB in primary cortical neurons [76]. This agonist was developed with the same strategy used for ogerin [37]. Silencing of *Gpr65* in human epidermal keratinocytes isolated from neonatal foreskin also blunts the cAMP induction by acidification via hypercapnia [112]. As a consequence, this silencing reduces phosphorylation of IκBα and p65 and elevates IL6 and TNF production [112]. As for OGR1, receptor internalization was observed for TDAG8 in HEK293T cells after acute stimulation with low pH [39].

## GPR4

GPR4 also stimulates cAMP formation and protein kinase A activation via Gα_s_ [75]. However, its signaling shows diverse modalities. For example, GPR4 can couple to Gα_q/11_ and Gα_12/13_ [58, 122, 142], or to Gα_s_, but activating EPAC instead of PKA [10]. By large, research on GPR4 signaling has been performed in immortalized cell lines but much less is known about its signaling in vivo or ex vivo conditions and its regulation. Collectively, these studies show an influence of this receptor on multiple downstream effectors and pathways, such as the hippo pathway, MEK/ERK signaling, and activation of SRE and NFAT [99, 122, 154]. There are no known pharmacological agonists for GPR4, but several antagonists have been developed and successfully applied in vivo [25, 82, 109, 129, 137].

In summary, all three GPCRs can signal via different Gα subunits and coupling may occur in a cell-specific manner. Moreover, the sensitivity to pH may also dependent on the Gα subunit associated to the respective GPCR [52] (Fig. 1).

## Interactions among proton-activated receptors and with other receptors

Little is known about physical or functional interactions between proton-activated receptors and other receptors. As mentioned above, OGR1 and G2A can form heteromers if cotransfected [39], OGR1 and GPR4 can also form homomers, and OGR1 and TDAG8 may heteromerize [39]. Whether any of these dimers occur in vivo and have functional relevance remains to be established given that cellular expression patterns between these receptors differ and that many GPCRs are arranged by scaffold proteins that cluster proteins in distinct cellular domains. It has been also suggested that GPR4 may form heterodimers with LPA and S1P receptors [157]. However, these findings were later criticized, given that GPCRs may mostly heteromerize within members of the same subfamily [22]. However, reciprocal functional regulation between OGR1 and the calcium sensing receptor (CaSR) was demonstrated by Wei et al. in primary cerebellar granule cells [139] where stimulation of OGR1 inhibited CaSR and vice versa. Moreover, conditions that stimulate each receptor, i.e., reduction of extracellular pH for OGR1 and elevation of extracellular calcium for CaSR, inhibit the signaling dependent on the other receptor. Interestingly, another study performed by Huang et al. with HEK293T cells showed that extracellular calcium had a small stimulatory effect on OGR1 [38]. The discrepancy might be explained by endogenous CaSR expression in HEK293T [33, 133]. When Wei et al. inhibited CaSR with specific antagonists, low pH caused an increase in intracellular calcium via OGR1 in cerebellar granule cells [139].

Given that certain cell types express more than one member of the subfamily of proton-activated GPCR, one could expect the existence of functional coordination between them [32, 46]. Limited expression data obtained from *Gpr4*, *Gpr65*, or *Gpr68* knockout models have not shown changes in the mRNA expression of the other two members when one was deleted. However, there are multiple examples of OGR1 and TDAG8 performing opposite roles especially in immune cells during inflammatory processes (see below section “A brief overview on the role of proton-activated GPCR in organ inflammation”) , but it is unknown whether this originated from functional coordination. However, an example of functional complementation was observed between proton sensors of different protein families: GPR4 and TASK2, a potassium channel activated by protons, in stimulating respiration in the retrotrapezoid nucleus (see next section *GPR4 and OGR1 in control of breathing)*. Another similar example is observed in ipsilateral joints suffering from rheumatoid arthritis, in which three proton sensors contribute to inflammation and pain: TDAG8, transient receptor potential vanilloid subtype 1 (TRPV1), and acid-sensing ion channel 3 (ASIC3) [36].

## GPR4 and OGR1 in control of breathing

The pattern of breathing is highly sensitive to pH [29, 118]. In mammals, central and peripheral chemosensors are responsible for the detection of alterations in O_2_, CO_2_, and pH and communicate these changes to central areas responsible for controlling and generating breathing patterns. Peripheral CO_2_ sensing is accomplished by glomus cells in the carotid body. Unpublished data from our group suggest that this structure expresses GPR4 but its contribution to respiratory regulation remains unknown. The retrotrapezoid nucleus (RTN) is a brainstem area containing chemosensitive neurons that are excited by extracellular acidification [29]. RTN neurons from WT mice showed an increased depolarization and firing rate when exposed to acidic pH, whereas RTN neurons from *Gpr4* knockout mice exhibit low sensitivity to CO_2_ and acidic pH stimulation [61]. The exact signaling mechanism and targets of GPR4-dependent signaling have remained elusive to date. NE 52-QQ57, a GPR4 antagonist, reduces the hyperventilatory response to hypercapnia [35]. Likewise, mice lacking *Gpr4* show a reduced ventilator response to increasing concentrations of CO_2_ in the ambient air while mice deficient for either *Gpr68* (OGR1) or *Gpr65* (TDAG8) have normal respiratory responses [61]. Genetic rescue experiments reintroducing *Gpr4*, specifically in RTN neurons, mostly restore the respiratory defect in mice suggesting that GPR4 is both required and sufficient to drive CO_2_-dependent changes in respiration. Interestingly, mice deficient for TASK2, a potassium channel present in a subset of RTN neurons and also activated by protons, exhibited a similarly reduced response to pH alterations and GPR4/TASK2 double knockout mice show an almost completely abrogated response to CO_2_ and pH variations with respiratory failure and high lethality after birth [61]. This work demonstrates that GPR4 is not only important for breathing control, but also that within a specific organ pH sensors may work in a redundant and complementary fashion.

In brain, GPR4 is not only found in RTN neurons but is also highly abundant in the endothelial cells of the brain vasculature [35, 142]. Both metabolic and respiratory acidosis elevate cerebral blood flow in most but not all brain regions [23, 24]. In mice, CO_2_ induces vasoconstriction in the brainstem, the site where RTN neurons and the centers regulating breathing are located. In contrast, in the same animals, CO_2_ caused vasodilatation and increased blood flow in the amygdala. Both vasoconstriction in the brainstem and vasodilatation in amygdala were attenuated in mice with global GPR4 deletion [142]. A similar reduced vasoreactivity to CO_2_ was found in mice with brain-specific deletion of Gα_q/11_, the Gα protein likely mediating GPR4 signaling in endothelium. Reduced vasoreactivity in Gα_q/11_ KO mice was paralleled by an impaired respiratory response which was less pronounced than in mice globally lacking GPR4 and suggested that endothelium plays an important role in the brainstem response to elevated arterial CO_2_. The authors hypothesized that CO_2_-induced vasoconstriction serves to accumulate CO_2_ in brainstem regulating RTN neuron activity. Gα_q/11_ KO mice showed also increased CO_2_-induced anxiety behavior suggesting that endothelium-dependent regulation of blood flow in amygdala contributes to fear reactions [142]. The difference in CO_2_-induced vasoreactivity between vessels of the brainstem and amygdala may be related to a reduced CO_2_-stimulated release of the vasodilators NO, PGF1α, and PGE2 from brainstem-derived endothelia.

Thus, GPR4 modulates CO_2_/pH-dependent breathing through at least two distinct mechanisms by either directly regulating RTN neurons or by inducing vasoconstriction in brainstem. The latter is possibly enabled by the absence of vasodilating effectors. As discussed below, GPR4 may also play a role in kidney in modulating the renal response to acidosis and by increasing the capacity of the kidneys to excrete acids. Functioning both in the respiratory and renal responses to an acid load, GPR4 may take a central role in the defence mechanisms maintaining systemic acid–base balance. This might also limit the use of systemically acting GPR4 antagonists.

In addition, pH sensing via OGR1 also influences contractility of smooth muscle cells and has a direct impact on airway resistance [111]. The role of OGR1 in airway physiology was recently reviewed by Nayak and Penn [90]. Briefly, in vitro studies demonstrated that OGR1 can cause contraction or relaxation of airway smooth muscle cells depending on whether it signals via Gα_q_ or Gα_s_. Biased signaling with different benzodiazepines activates (agonism) either both or a single G protein type (Gα_s_). This observation expands the range of possibilities for targeted therapies, which might also be extrapolated to other organs. However, the therapeutic potential of OGR1 controlling airway resistance has still to be demonstrated in vivo.

## Proton-activated GPCR in brain function

Brain acid–base status modulates key parameters to brain function like cerebral blood flow, brain metabolism, and neuronal activity [45]. All proton-activated GPCRs are expressed in the brain and have been recently implicated in some of these functions in health and disease [35].

Hypercapnia is also a potent inducer of certain behavioral activities, such as fear, anxiety, and panic [21]. Acidification by high PCO_2_ alters neuronal activity and stimulate proton sensors in multiple areas of the brain associated with these behaviors [131]. In vivo and ex vivo experiments with mice or murine brain slices exposed to hypercapnia in vitro demonstrated that low pH in the subfornical organ is detected by TDAG8 in the microglia, which induces the release of the cytokine IL1β [130]. This cytokine stimulates neurons of the subfornical organ, which finally communicates with effector areas responsible for cardiovascular, freezing, and fear responses. Patients with panic disorders show an (modest) elevation of *Gpr65* (TADG8) expression levels in peripheral blood mononuclear cells in comparison with control individuals [115]. Moreover, mice lacking TDAG8 seem to show reduced anxiety and depression after a forced swim test [80]. As aforementioned, activation of OGR1 with the agonist ogerin inhibits fear conditioning [37]. Interestingly, another group reported that OGR1 knockout mice have disturbed hippocampal synaptic activity, which leads to impaired avoidance memory [149].

Brain ischemia, a common condition to multiple diseases and disorders, also imposes alterations to brain acid–base balance. Different severities of acidosis and alkalosis may occur from acute to subacute phases of ischemia [66, 124] and changes in brain extracellular and intracellular pH values have been associated to additional injury or neuroprotection [123]. It has been proposed that mild acidosis is neuroprotective, but in severe ischemia when pH can fall to values around 6, acidosis exacerbates the damage [53, 123]. Indeed, mild acidosis in mice activates OGR1 and provides neuroprotective effects after transient middle cerebral artery occlusion [135]. Mice lacking OGR1 showed enlarged infarct area and performed worse in behavioral tests. Conversely, bicarbonate injection in the injured area and *Gpr68* overexpression in the brain attenuated the damage [135]. Authors speculated that in mild acidosis, PKC activation via OGR1 provides protective effects, while in severe acidosis, other protons sensors, such as ASICs and the proton-activated chloride channel (PAC), are active and cause further damage. RNA sequencing data from brains collected from Gpr68 knockout mice subjected to transient middle cerebral artery occlusion do not show changes in the expression of ASICs or PAC [158]. However, *Gpr68* deletion caused changes in three genes encoding hemoglobin and a few genes already associated with neuroprotection. In a similar manner, activation of TDAG8 has also been proposed as neuroprotective in brain ischemia [76]. The TDAG8 agonist BTB09089 reduced infarct area in ischemic mice and also the expression of inflammatory markers 24 h after reperfusion.

On the other hand, pharmacological GPR4 inhibition prevented cognitive impairment in mice receiving the neurotoxin 1-methyl-4-phenyl-1,2,3,6-tetrahydropyridine (MPTP), a Parkinson’s disease model [31]. GPR4 might prevent apoptosis and the reduction in the number of dopaminergic neurons at least in the striatum and substantia nigra pars compacta induced by MPTP. Therefore, agonists and antagonists of proton-activated GPCR may be promising tools to treat diseases and conditions that cause pH-mediated injury to the brain or to stimulate neuroprotective mechanisms.

## GPR4 and OGR1 and renal function

As a normal response to an acid load, the kidneys increase urinary acidification and  the synthesis of new bicarbonate mainly through the process of ammoniagenesis and by excreting higher amounts of ammonium (NH_4_^+^). Also, several urinary buffers known as titratable acidity (mostly phosphate, but also citrate, urate, and creatinine) are excreted [132]. These processes are coordinated and regulated both not only by systemic factors such as angiotensin II, aldosterone, or endothelin but also by local mechanisms induced by local acid–base sensors [5]. *Gpr4* knockout mice show a more alkaline urine with lower urinary titratable acidity under baseline conditions and lower NH_4_^+^ excretion when subjected to an acid load for four days paralleled by an incomplete adaption of acid–base transport proteins in the collecting duct [116, 117]. Chronic acidosis leads to a remodeling of the collecting duct with a relative increase in the number of acid-secretory type A intercalated cells, which in part is mediated by proliferation of these cells triggered by GDF15 [41, 141]. GPR4 is expressed in type A intercalated cells at very low level and at a higher levels in neighboring principal cells. Principal cells secrete GDF15 in response to acidosis which then stimulates type A intercalated cell proliferation. GPR4 is not required for acidosis-induced GDF15 secretion but for its action on type A intercalated cells [12]. Whether the link between GPR4 and GDF15 extends beyond the kidney remains to be addressed.

OGR1 has also been implicated in the control of urine acidification. Renal HEK cells transfected with OGR1 show higher sodium dependent and independent proton secretion capacity while this response was not seen in cells transfected with OGR1 lacking critical histidine residues [85]. However, counterintuitively, isolated proximal tubules from *Gpr68* (OGR1) knockout mice show an elevated proton secretion rate [85]. Consistently, apical brush border membrane preparations from *Gpr68* knockout mice subjected to 7-day NH_4_Cl load show increased expression of the proton secreting protein sodium hydrogen exchanger 3 (NHE3, *Slc9a3*) [44]. The typical elevations in ammonium and calcium excretion by chronic metabolic acidosis are also disturbed in *Gpr68* KO mice [44]. Gpr68 KO mice do not develop the hypercalciuria typical for metabolic acidosis which may be caused by the higher expression of NHE3 in the proximal tubule driving increased paracellular Ca^2+^ reabsorption in this segment. Also, the expression of the TRPV5 Ca^2+^ channel, present in the late distal convoluted tubule and connecting tubule, was enhanced in acid-loaded *Gpr68* KO mice compared to their littermates. Surprisingly, *Gpr68* mRNA expression is barely detectable in all these nephron segments while it can be detected in interstitial renal cells raising the question how Gpr68 may influence these tubular transport processes.

## Proton-activated GPCRs in bone

Extracellular pH is a strong modulator of bone structure and physiology. Chronic metabolic acidosis impairs bone mineralization, which was initially considered to be mostly a consequence of physicochemical dissolution of the mineral constituents of bones [3, 26]. However, pH is a key regulator of osteoblastic and osteoclastic activities and the physicochemical effect is considered only a minor component [3]. Therefore, it is conceivable that bone cells require pH-sensing mechanisms for the response to local or systemic acid–base changes. Indeed, proton sensors like ASIC1 and TRPV1 play important roles in pH-mediated bone functions [70, 103]. All three receptors are expressed in bone cells. GPR4 has been found in osteoblasts [93], OGR1 has been detected in osteoclasts [152] and osteoblasts [75], and TDAG8 has been shown in osteoblasts [93] and in osteoclasts [34].

Little is known about the role of GPR4 in bone; a role in osteoblast synthesis of the receptor activator of nuclear factor-kappa B ligand (RANKL) has been shown in vitro, while the in vivo relevance is unclear [93]. In contrast, the role of OGR1 in bone has been studied by several groups, however, with conflicting results. In vitro, suppression of OGR1 with siRNA inhibits osteoclastogenesis [98, 152]. At least four different studies reported apparently contradicting phenotypes in the bones of OGR1 knockout mice [44, 59, 67]. Krieger and colleagues identified increased osteoclast activity along with higher bone turnover and mineral density in 8-week male mice with global OGR1 deletion [60]. However, studies from the same group showed that osteoclast-specific OGR1 deletion in 10–12 weeks female mice causes the opposite effect, a lower osteoclastic activity [59]. In this work, authors did not observe differences in bone microstructure in male mice. Li et al. also investigated bones of 8-week-old mice with global deletion of OGR1. For this, they examined two pairs of homozygous floxed control and OGR1 knockout animals, one female and one male per pair. They did not find any major abnormality in bones [67]. Still, they reported reduced osteoclastogenesis when using peritoneal macrophages induced with RANKL. We have also investigated the bones of 16-week-old OGR1-deficient mice and we did not find microstructural abnormalities at baseline and after 4 and 8 weeks of acid loading with NH_4_Cl in both male and female mice [44] using the same model as Krieger et al. in [60]). Around postnatal week 16, skeletal growth in mice is completed, which suggests that OGR1 might have a more prominent role in earlier periods. However, we also examined osteoclastic activity in vitro from non-adherent bone marrow cells stimulated with macrophage colony-stimulating factor and RANKL [44]. Cells were collected from 6- to 8-week-old male and female mice, and we did not find any relevant functional difference between both genotypes. Osteoclastic-specific OGR1 deletion as generated by Krieger et al. is an important step to dissect the role of OGR1 in bone independent of other non-osseous functions. In humans, rare homozygous mutations in OGR1 have been found in three families with amelogenesis imperfecta, a rare disease impairing mineralization of tooth enamel [96]. OGR1 was detected in enamel and mutations detected are a frameshift, an in-frame deletion, and a missense mutation which impairs pH-dependent activation of OGR1 [110] suggesting that OGR1 has an important role in tooth development and mineralization.

Also, TDAG8 (*Gpr65*) is expressed in bone, mostly in osteoclasts. In ovariectomized mice, bone resorption was enhanced in the absence of TDAG8 along with a higher number of osteoclasts and increased osteoclast activity [34]. These data suggest that TDGA8 may exert a suppressive function in osteoclasts and that its absence causes excessive bone resorption.

## OGR1 and GPR4 and insulin secretion and sensitivity

Acid–base balance also modulates endocrine functions. In humans, metabolic acidosis consistently causes insulin resistance while alkalosis has opposite effects by mechanisms not well understood yet [79, 145]. In vivo experiments demonstrated an increase in glucose stimulated-insulin release by acidosis and a decrease by alkalosis [88, 126]. However, experiments in vitro with isolated pancreatic islets produced diverse results, with either a stimulation by medium alkalinization [71] or inhibition/no effect by both low or high pH [42, 83]. OGR1-deficient mice show baseline reduced insulin and glucagon levels, while keeping normal blood glucose levels [88]. Additionally, these mice do not exhibit an elevated glucose-stimulated insulin secretion in response to acidification, and OGR1-deficient isolated pancreatic islets subjected to low pH conditions do not secrete insulin. Caution should be taken when analyzing these data, as most of the previous studies with isolated pancreatic islets have shown an inhibition or lack of effect by acidosis in glucose stimulated-insulin release instead of stimulation [42, 71, 83].

In contrast to the phenotype observed in OGR1 KO mice, mice lacking GPR4 have lower fasting glucose levels with inappropriately normal to high insulin levels suggesting increased insulin sensitivity [28]. Indeed, GPR4 is highly expressed in white adipose tissue and mice lacking GPR4 showed faster return of glucose levels during the intraperitoneal glucose tolerance test and lower glucose levels when injected with insulin. In the absence of GPR4, mice had also higher circulating leptin levels while expression of PPARα was decreased in the liver, skeletal muscle, and white adipose tissue. Interestingly, in mice fed a high fat diet, the differences in glucose metabolism mostly disappeared between genotypes while in aged mice, the absence of GPR4 was still associated with increased insulin sensitivity The exact mechanisms on how GPR4 interferes with cellular insulin signaling, however, remains to be established.

## Gastrointestinal tract

Substantial work has been done investigating the role of proton-activated GPCRs in inflammatory diseases of the gastrointestinal tract because of their potential role in other inflammatory diseases and the identification of single nucleotide polymorphisms (SNPs) in and close to *GPR65* that associate with inflammatory bowel disease in several cohorts [1, 74]. We briefly cover this topic in the next section, but interestingly, not much is known regarding the roles of these receptors in normal intestinal functions. TDAG8 is a marker of a subset of vagal afferents innervating intestinal villi [9, 144]. These neurons are involved in the detection of nutrients and regulation of intestinal motility, but proton sensitivity was not investigated in this context [144]. Proton sensors may also be modulated by the proton pump inhibitor omeprazole, which is a widely used drug in treatment of gastroesophageal reflux disease, peptic ulcer disease and other diseases affecting the gastrointestinal tract. When omeprazole is used to treat Caco-2 cells, an immortalized cell line commonly used as a model of intestinal epithelia, the expression levels of proton sensors ASIC1a, TRPV4, and OGR1 are altered [121]. Inhibition of OGR1 with Cu^2+^ or an OGR1 neutralizing antibody reduced magnesium transport in these cells [121]. However, the quality of these antibodies is unclear and copper inhibits many cellular processes at the concentrations used in this study.

## A brief overview on the role of proton-activated GPCRs in organ inflammation

Even though the role of the proton-activated GPCRs in pathological states is not the main topic of this review, we will briefly mention some major findings as this may be instructive to understand their roles particularly in the immune system. The co-occurrence of inflammation, hypoxia, and local acidification has been known for a long time, but the role of pH as a modulator of pro and anti-inflammatory pathways has been only more recently recognized [81, 104, 105]. In addition, local inflammation and concomitant hypoxia are themselves additional factors causing local acidification [20]. Activation of proton-activated GPCRs has been implicated in the regulation of inflammatory processes that span from the infiltration of immune cells to their differentiation, proliferation, and activity [7, 27, 120, 136, 153]. The abundant expression of GPR4 in endothelial cells allows to mediate at least in part the pH-dependent activation of endothelial cells and subsequent immune cell invasion [58, 92]. GPR4 activation by low pH increases vessel permeability by increasing paracellular formation of gap junctions [58]. Moreover, GPR4 has also been implicated in endoplasmic reticulum stress and inflammation in endothelial cells in vitro [15, 16]. Among immune cells, *Gpr4* mRNA has been detected in B cells by single cell RNA sequencing, and it might be expressed in monocytes and macrophages but its function in these cells has not been examined in detail [65, 84, 95]. OGR1 and TDAG8 are found in multiple immune cell types, such as macrophages, monocytes, dendritic cells, neutrophils, T and B cells, and natural killer cells [2, 86, 95, 128, 146, 151]. In addition, TDAG8, which is the most abundantly expressed proton-activated GPCR in immune cells, is also found in eosinophils and mast cells [56, 146, 162]. Other cells of importance for inflammatory processes also express proton-activated GPCRs, such as epithelial cells, fibroblasts, and smooth muscle cells. Therefore, a simplified view of the organization of the these receptors in immune responses would be that TDAG8 and OGR1 control pH-dependent activities (e.g., proliferation, cell activity) in various immune cells, while GPR4 plays rather a role in activation of endothelial cells and facilitation of infiltration. Whether OGR1 also contributes to the vascular response during inflammation has not been addressed to date. However, one should also take into consideration that these receptors also play important roles in other cell types involved in inflammatory processes (and associated events) like epithelial cells, fibroblasts, and smooth muscle cells [77, 90, 111, 137]. For example, OGR1 has been shown to play a role in endoplasmic reticulum stress in an epithelial cell line [77], inhibition of GPR4 in fibroblasts reduces pH-dependent transition to myofibroblasts [137], and TDAG8 participates in proliferation and migration of smooth muscle cells with impact in atherosclerosis [11].

Large part of the current knowledge on the role of these receptors in inflammation comes from studies on gastrointestinal and lung inflammation, and experimental autoimmune encephalomyelitis (EAE) [2, 18, 40, 125, 136, 146]. While the experimental strategies were very diverse in these studies, there were some common findings across tissues and organs. OGR1 deficiency was mostly anti-inflammatory and protective while TDAG8-deficiency caused opposite effects [2, 18, 40, 120, 125, 146]. We will term this here as “OGR1-TDAG8 reciprocity,” but it still unknown whether this phenomenon is relevant and whether it occurs in an independent or coordinated manner. In inflammatory bowel disease models, genetic and pharmacological inhibition of GPR4 was also protective and a promising target for therapies aiming at reducing intestinal fibrosis [109, 136, 137]. Pharmacological inhibition of OGR1 also produced similar protective effects [127]. On the other hand, biased agonism via benzodiazepines might provide a different angle for the treatment of asthma and other inflammatory diseases [89].

While we do not cover in this review the role of proton-activated GPCRs in tumor biology, this topic has already been reviewed by others [49, 140]. Cancer cells commonly have elevated intracellular pH compared to normal cells while often the extracellular milieu around the tumor is more acidic. Inflammatory processes modulated by pH are often very important in tumors and pH-sensing mechanisms might become valuable targets for the treatment of cancer.

## Open questions

There are multiple open questions that should be addressed and we like to discuss some of them here. There are also technical and/or biological issues that have been hampering this research field and may require special attention.

1. Functional properties: Even though there are a few GPCRs that couple to several Gα proteins, proton-activated GPCRs seem to signal through cell- and maybe even context-specific Gα proteins. Attention may be needed to determine the specific signaling pathway used by specific receptors.
2. The field is still hampered by the lack of reliable and sufficiently specific antibodies allowing for the exact localization of these receptors or performing studies on their regulation. Proton-activated GPCR reporter mice have been used to partly circumvent this issue [137, 147].
3. Cell or organ specific knockout models may be a helpful tool to avoid multiple confounding factors observed in global knockout models.
4. Use of specific agonists and antagonists for each receptor is needed and is becoming now available. The use Cu^2+^ or Zn^2+^ as inhibitors is problematic as these metals interfere not specifically with a single type of proton-activated GPCR and also react with many other cellular proteins.

These tools and precautions will be useful to address open biological questions:

- No genetic buffering/redundancy among proton-activated GPCR has been observed in data coming from studies with knockout mice (e.g., deletion of GPR4 does not lead to a compensatory increase in OGR1 expression). Is there functional complementarity coming from non-GPCR proton sensors? TASK2 and GPR4 functional complementarity [61] illustrates such type of interaction. Double/triple knockout models or concomitant pharmacological inhibition may reveal potential functional interactions and compensatory effects.
- Which RGS proteins are involved in the regulation of these receptors? How are proton-activated receptors otherwise regulated? Cellular models are needed, but these studies may be complicated by the fact discussed above that cell type–specific mechanisms may exist.
- Is the mechanosensing activity described for OGR1 [138, 147] a general OGR1 feature in cell types other than those previously tested? Do other proton-sensing GPCRs also show mechanosensing activity?
- In some cell types, OGR1 and TDAG8 show functional “reciprocity.” Are they regulated in concert? Does this relationship have a relevant biological meaning? Again, cell and animal models with deletion of two or all receptors may be helpful to address some of these questions
- Which other well-described biological processes modulated by extracellular pH are regulated by these proton-activated GPCRs? (e.g., renal ammoniagenesis, hepatic urea production, muscle proteolysis, brain energy metabolism, immune cell chemotaxis)
- What is the molecular identity and role of other pH-sensing mechanisms and how do these mechanisms interact with the proton-activated GPCRs?
- Are proton-activated GPCRs relevant drug targets in human disease and are drugs targeting them safe for humans? Given the ubiquitous expression of these receptors, drugs may need to be administered locally or be delivered in a target-specific manner, e.g., only topical as inhalation or non-absorbable drug.

## Summary

**Fig. 2:**
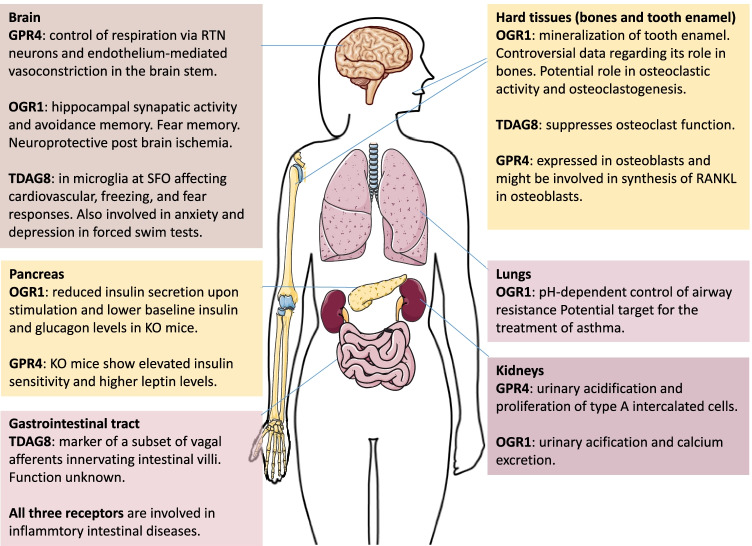
Summary of the main physiological roles of proton-activated GPCR. RTN, retrotrapezoid nucleus; SFO, subfornical organ; RANKL, receptor activator of nuclear factor-kappa B ligand

Survival of cells relies on the constant detection of intra- and extracellular alterations and on the capacity of adapting to environmental changes. Although multiple cellular phenomena caused by alterations in pH levels have been described over the last century, the identification and investigation of proton sensing mechanisms provided some missing key elements for the understanding of multiple biological questions that are regulated by proton-activated GPCRs (Fig. 2). Proton-activated GPCRs are cell membrane receptors enriched in amino acids that can be protonated or deprotonated in a pH range compatible with the pH found in most extracellular biological fluids (~ 6–7.4) and thereby elicit intracellular signaling via a variety of Gα subunits. While allosteric ligands (and in the case of OGR1, mechanostimulation) modulate the activity of these receptors, protons are considered the main ligand. Therefore, examining proton-activated GPCRs provides an opportunity to understand how pH governs multiple cell and organ activities without necessarily interfering with acid–base status of biological compartments. Pharmacological intervention at these receptors has been shown in preclinical studies to prevent detrimental conditions caused by diseases, but whether these drugs will be effective in and safe for humans are still central questions to be answered.
